# Institutional Notes and News

**Published:** 1910-01-22

**Authors:** 


### Institutional Notes and News.

**Leicester Infirmary.**

A quarterly meeting of the governors was held on Wednesday, the 12th inst., in the outpatients' hall of the infirmary (the board-room having been found too small for such gatherings), Sir Edward Wood presiding over a very large gathering. Several ladies and gentlemen having been elected life governors, the House Governor and Secretary submitted his report on the work of the year 1909, which showed that 2,608 inpatients had been admitted to the infirmary, 599 to the children's hospital (a grand total of 3,207); 12,573 new outpatients had been treated, and 30,131 had received renewed treatment, 12,336 new cases had been treated in the casualty department (of whom 354 had been admitted to the wards), and 1,097 cases had received treatment in the *x*-ray department. The daily average number of inpatients was 209.2. The receipts for the year had amounted to £22,822. Annual subscriptions show a total of £3,850, donations £1,056, income from investments £1,914, Hospital Sunday £2,175, Hospital Saturday £8,715 (the total amount received by this Fund was £12,850—a new record for Leicester being created), £950 was transferred from the children's hospital, and miscellaneous items brought in £221, a total ordinary income of £18,881. Legacies had amounted to £3,941, of which £1,000 had been invested in accordance with the known wishes of the testator, making a total income of £22,822 The expenditure on provisions had been £4,365, against £4,192 in 1908; surgery and dispensary £2,321, against £2,226; establishment charges £1,023, against £1,703; domestic £3,567, against £3,725; salaries and wages £5,425, against £5,159; miscellaneous £246, against £368; total £16,947, against £17,372. Management expenses had been £1,422, against £1,154; and other sundry expenditure £323, against £346; a grand total of £18,693, against £18,874. £1,000 had been invested (as *per contra*), which left a balance of income over expenditure for the year of £3,140, which had been applied, £400 for the provision of a reserve fund for future large expenditure on buildings, £2,726 in repayment of the deficit brought forward on January 1, 1909, so that there remained a small balance of £14 in hand. This statement was regarded by the governors as very satisfactory and as evidence of the increased interest which is being taken in the welfare of the institution.

**An Interesting Relic.**

Miss Sarah Jones, who died last week in the Royal Hospital for Incurables, Putney Heath, after being an inpatient of that institution for fifty-two years, left among her belongings the original of the following interesting letter :—

Buckingham Palace.

Major-General Sir T. M. Biddulph is commanded by the Queen to acknowledge the address of the invalid inpatients of the Royal Hospital for Incurables, Putney Heath, in which they offer to her Majesty and to the Princess of Wales their congratulations on the recovery of the Prince of Wales. Her Majesty fully appreciates the kind feeling which prompts them, and receives with more than ordinary gratification the good wishes of those whom it has pleased Providence to afflict, and who in their affliction so warmly participate in the general joy and thankfulness to God for His mercy to the Queen and to the nation.

February 26, 1872.

**Patients as Nuisances.**

A proposal was made at a meeting of the Guardians of St. George's, Hanover Square, to erect sound-proof walls to prevent the screams of patients escaping from the isolation block. Some of the members objected to the proposal, which, it was stated, would involve an expenditure of £200 or thereabout. Alderman Everitt pointed out that lunatics in the workhouse were under the Lunacy Commissioners, and ordinary sick patients were under the Poor Law, and the two classes could not be dealt with together. Padded rooms were used for lunatics in the workhouse, but these could not be used for sick people. If sound-proof walls would remedy the annoyance to any degree they ought to be constructed. The proposal was referred back to a joint committee of the Workhouse and Infirmary Committees.

**£20,000 Overdraft at Cardiff Infirmary.**

The Cardiff Infirmary Board of Management was in the unenviable position of announcing on January 12 that "the overdraft at the bank now amounted to £22,644." A letter from Colonel Bruce-Vaughan remarked that in spite of the fact that the income of the Infirmary had increased in 1909 by some £1,655, the expenses had increased to £2,300 more than that income. He gave as reasons for this (1) accumulation of overdraft by the amassing of annual deficits, (2) the extra cost and administrative expense of the new out-patients' department, (3) and the addition of 14 more beds costing £960. The Colonel's letter added that the extra work had been undertaken "because of the liberal response since 1906. Those who had given £79,000 in four years would surely provide for the annual upkeep." He suggested raising £700 to £800 a year by making out-patients pay a registration fee of 2d. per week, and in-patients 3s. per week towards the cost of their food. He proposed a personal appeal to every working-man, collier, and ratepayer, and that the churches and chapels should be approached. Such an appeal, he thought, should give the infirmary £2,000 a year. If something of the kind were not done £16,500 a year would be required, and when the new wing was opened at least £20,000 a year. An anonymous gift of £500 was reported. Lord Aberdare, Treasurer of the Infirmary, said that so long as the overdraft was kept below £20,000 he was bound to be satisfied. But he feared this year to see it mounting up to £24,000. A continual and increasing deficit of this kind is only too likely to make the withdrawal of legacies on the ground of extravagance, such as that reported in the newspapers recently, occur in more than one part of the country. We trust that Cardiff Infirmary will realise that a change of policy is advisable in the interests of its future local claims for support.

**A Presentation.**

At a friendly gathering in the Council Chamber a fortnight ago, Alderman C. J. Stoddart, Chairman of the Rotherham Hospital Committee, presented the Mayor (Councillor D. Mullins) with a silver rose-bowl, bearing the following inscription : "Presented by members of the Committee of the Rotherham Hospital to the Mayor of Rotherham (Coun. Dan Mullins), in recognition of his great services in raising the sum of £3,500 to pay off the debt on the hospital. 20th December, 1909." A year ago the Mayor inaugurated a fund for the purpose of paying off the debt on the hospital. So satisfactory was the response to his appeal that considerably more than the amount asked for was raised.

